# The Year's Work and War Prices

**Published:** 1918-01-26

**Authors:** 


### LEICESTER ROYAL INFIRMARY.

### The Year's Work and War Prices.

THE statistical reports of the large hospitals for the past year will afford exceptionally interesting study, and will be awaited with keen anticipation. There has never been a time when the work which they have accomplished has been so clearly recognised or met with a greater response from an augmented number of supporters. Several reasons can be advanced for this happier position. The Great War has undoubtedly had its influence in several ways. It has at a time of great national crisis proved the general efficiency of the voluntary institutions and their capability of undertaking a share of the national burdens. The difficulties of the individual in provisioning the home have certainly never been so acute as at present, and this has caused people to realise the onerous work of hospital administrators in the provisioning of large numbers of sick people. The high prices, which have been a primary reason for increased remuneration, have brought home to heads of families the knowledge that increased contributions are required by institutions. The various funds which have been promoted for war needs have been generously responded to, and have by their cumulative effect encouraged more generous giving. Not all institutions are, however, equally advantaged in the distribution of food supplies. Seaport towns handling large imports probably enjoy freer and more liberal supplies than the inland towns. Comparisons of statistics will, therefore, be of unusual interest this year.

From the yearly report of the work of the Leicester Royal Infirmary submitted to a meeting of the governors on Saturday, we learn that an income of approximately £41,000 was received, exclusive of legacies, and the gross expenditure was for practical purposes £40,000. Of this amount £11,000 was expended on provisions, and £10,600 on salaries and wages. The cost of equipping and maintaining the venereal diseases department was £2,880, which for the purpose of accounting is included under the head of Extraordinary Expenditure. The cost per occupied bed on the gross expenditure of the year works out at £124, a figure which, compared with pre-war years, shows an alarming increase. It is to be expected that similar increases will be shown by all institutions throughout the country.

### SUBSCRIPTIONS MORE THAN DOUBLED.

The income exhibits several encouraging features. A great effort by Mr. Alfred Corah, the treasurer, during the year of his shrievalty of the county, increased the subscription list in 1916 from £4,184 to £8,254. This amount has expanded in the past year owing to sustained efforts on Mr. Corah's part, and now stands at £8,700. £3,000 of this sum is deducted from the income account and set aside as a reserve fund for future needs and extensions, in accordance with the wishes of the treasurer, who seeks to avoid further appeals for capital outlays. Donations, after deduction of amounts received for endowments, have expanded from £2,800 to £3,560, church collections from £2,234 to £2,500, and miscellaneous receipts, including amounts received for maintenance of military patients, stand at £16,256. The returns of the Saturday Hospital Society, as yet incomplete, show an estimated total of £17,500, compared with just under £17,000 in the previous year. Of this sum the infirmary receives a seven-tenths proportion. The gross ordinary income shows an increase of £8,500 to set against an increased expenditure of £7,800.

### THE CHAIRMAN'S REPORT.

The Chairman, Mr. T. Fielding Johnson, junr., J.P., reported that the memorial to Sir Edward Wood, the rebuilding of the remaining unreconstructed wing, and the provision of thirty additional beds was being responded to in a promising manner, £7,500 having been provided towards the £25,000 required. He referred to the withdrawal from treatment in the military hospitals of discharged sick and wounded soldiers, and indicated that it would be a serious hardship further to reduce the number of civilian beds in the voluntary institutions, which had already liberally responded to the call for beds for wounded and sick soldiers. The report called attention to the increasing difficulty and rapidly increasing cost of obtaining food supplies, and stated that for practical purposes one-third of the ordinary income was now required for the purchase of provisions. The number of endowed beds and cots had been increased by six, and the report expressed a hope that it might become generally recognised that a most useful and public-spirited method of perpetuating the names of town and county residents was by the endowment of beds in the institution. In connection with Tank Week in the town several contributions of War Bonds had been received, one for £100 being in recognition of treatment afforded in the Children's Hospital fourteen years ago. The report paid a tribute to the services of the honorary staff, the resident medical officers, the matron, sisters, and nursing staff, and the administrative staff, who had rendered valued assistance in a trying year. The work of the institution had been on an increased scale; 4,400 in-patients had been received, as compared with 4,004 in the previous year. The daily average number of beds occupied had been 320, as compared with 275. Attendances in other departments in the years 1917 and 1916 had been: Out-patients, 19,727 and 18,163; accidents, 46,730 and 40,035; *x*-ray department, 3,965 and 2,823; operations, 4,413 and 4,697. In the pathological department the appointment of a whole-time officer had been amply justified, and 2,939 reports on various materia submitted had been made. The orthopædic department had given treatment on 6,102 occasions.

The report was adopted on the motion of the Treasurer, seconded by the High Sheriff (Mr. Hugh Goodacre).

